# Supplementary material for: NCBI’s Virus Discovery Hackathon: Engaging Research Communities to Identify Cloud Infrastructure Requirements
Source: Genes (Basel). 2019 Sep 16;10(9):714. doi: 10.3390/genes10090714 (PMC6771016; doi:10.3390/genes10090714)
Supplement: Supplementary file 1 [file genes-10-00714-s001.pdf]

Supplemental Tables

Supplemental Table 1

Tool	Version
BLAST	
BLAST databases	
SRA toolkit	2.9.4 (pre-release)
HISAT2	2.9.2
SEKSA	2.2
guidedassembler_graph	v2.0
compute_coverage	2.9.0
Python	2.7.15+ / 3.6.7
C++	6.3.0
R	3.3.3
Anaconda2	1.7.2
Conda	4.5.11
Bedtools	2.26.0
GATK	3.8-1-0
Picard	2.8.17
BWA	0.7.17
MiniMap2	2.14
Bowtie2	2.3.0
EDirect	N/A
HMMER	N/A
Samtools	1.9
BCFTools	1.9
HTS JDK	≥ 2.18.0
HTS lib	1.9
STAR	2.6.0
Abyss	≥ 2.0.2
plink-ng	v2.0
Cufflinks	2.2.1
Cytoscape-impl	3.7.0
Velvet	1.2.10
Tophat	2.1.1
FastQC	0.11.8
HTSeq	≥ 0.11.0
mcl	≥ 14-137
MUSCLE	≥ v3.8.31
MrBayes	3.2.6

GARLI	≥ 2.1
Clustal omega	1.2.4
Dedupe	≥ 1.9.4
TrinityRNASeq	2.8.4
Jupyter	4.4.0
Bioconductor-deseq2	≥ 1.20.0
jModelTest	N/A
IGV	N/A

Supplemental Table Legends

Supplemental Table 1: Pre-installed tools. NCBI staff prepared a VM image using GCP tool, such that the listed tool were pre-installed.
